# Doing the Right Thing: Caregivers and Medical or Nursing Tasks in the Post-Acute Home Care Setting

**DOI:** 10.1093/geroni/igaa057.811

**Published:** 2020-12-16

**Authors:** Jo-Ana Chase, David Russell, Daniel Kaplan, Michael Bueno, Rungnapha Khiewchaum, Penny Feldman

**Affiliations:** 1 University of Missouri, Columbia, Columbia, Missouri, United States; 2 Appalachian State University, Boone, North Carolina, United States; 3 Adelphi University, Cortlandt Manor, New York, United States; 4 Visiting Nurse Service of New York, New York, New York, United States

## Abstract

Family caregivers often manage complex medical/nursing tasks (MNTs) for older adults returning home after a hospitalization. The purpose of this qualitative study was to describe caregivers’ experiences leveraging diverse resources to manage MNTs for older adults receiving post-acute home health care services (HHC). In-depth telephone interviews were conducted with 20 caregivers of older adults who received HHC following hospitalization. Interviews were digitally audio-recorded, transcribed, and analyzed using directed content analysis. The Theory of Dependent-Care informed the analytical framework. We organized codes using three theoretical constructs related to managing older adults’ MNT care needs (“tasks related to the other”), accessing existing social/environmental resources (“tasks related to the situation of care”), and working with the healthcare system (“tasks related to the system of care”). Caregivers’ descriptions of MNTs included the complexity and socioemotional impact of assisting in these tasks (e.g., dependency, trust). When needed, caregivers’ accessed social (e.g., family, friends) and environmental (e.g., neighborhood, housing) resources to help address the older adults’ care needs. Caregivers also identified challenges and strategies for navigating and coordinating care and services within HHC and the larger healthcare system. Caregivers assisting with complex MNTs in the post-acute HHC setting need additional training and support. HHC providers can actively engage caregivers by tailoring training and support strategies, assessing social and environmental contexts and resources, and facilitating caregivers’ navigation of the healthcare system. Future research could elucidate social and environmental factors associated with successful collaborative relationships among providers, older adults and their caregivers in the post-acute HHC setting.

